# What Do I Want from the Publisher of the Future?

**DOI:** 10.1371/journal.pcbi.1000787

**Published:** 2010-05-27

**Authors:** Philip E. Bourne

**Affiliations:** Skaggs School of Pharmacy and Pharmaceutical Sciences, University of California San Diego, La Jolla, California, United States of America; European Bioinformatics Institute, United Kingdom

When I took on the role of Editor-in-Chief of this open-access journal, I began, for the first time, to think about scholarly communication beyond submitting my papers and getting them published. This thinking led to previous Perspectives [1]–[3], all of which shared an underlying theme—there are many opportunities to achieve better dissemination and comprehension of our science, and as producers of that output I believe authors have a responsibility to see it used in the best possible way.

No need to take my word regarding the opportunities that exist to improve scholarly communication and comprehension. I recommend reading “Part 4: Scholarly Communication” from the free online book the *Fourth Paradigm: Data Intensive Scientific Discovery* [4] (http://research.microsoft.com/en-us/collaboration/fourthparadigm/), which is a tribute to the late Turing Award winner Jim Gray. Jim, and many of the authors who pay homage to his vision, have thought deeply about the subject of scholarly communication. They conclude that data and knowledge-driven computation is indeed a fourth wave, as computation has impacted science to the point where every aspect of it is touched by computation (hence the name eScience), including dissemination and comprehension. These visionaries recognize that we are at a tipping point at which scholarly communication will change from a traditional print-oriented medium (albeit an online version of the print journal) to something else. That something else begins to transform today's research article as we realize the power of the medium, establish new forms of knowledge discovery, and measure the impact of scholarly contributions in new ways. For all that vision, these luminaries do not address the question that I have been pondering, and which I would like to raise here. Assuming all this innovation takes place, what will the publisher of the future look like, and as a contributor and consumer of a publisher's services in this new era, *what do I want from the publisher of the future?*

Recently, at gatherings of publishers where I have been invited to speak, I have been trying to pose and then answer this question. Unfortunately, I fear that what I propose appears so radical as to be greeted with either blank stares or looks of *get real*. Let me try here to do a better job at stating what I want from my publisher in the future.

Many of you are undoubtedly thinking that just accepting your papers will be enough, but bear with me. Presumably, publishing will continue in the life sciences (unless we go over completely to an ArXiv.org or similar model where articles are simply deposited without peer review and impact measured by how much they are accessed), and if so, will continue to be overseen by the publishers we, as scientists, work with today. A few new and innovative publishers like the Public Library of Science (PLoS) will continue to emerge as business models and practices change, but existing publishers will probably adapt in this new era. I anticipate similarities to earlier phases of the Internet revolution. Amazon.com emerged as a new and major online-only shopping entity, but Sears, Wal-Mart, Harrods, etc., while being slower in adopting the new medium, did eventually successfully support online shopping and a range of new services. By comparison, a few innovators have had some impact on scholarly communication, but traditional science, technology, and medical (STM) publishers will continue to dominate the conservative and relatively slow-moving market. These pioneering publishers are now experimenting with interactive PDFs, “articles of the future,” semantic tagging, data integration with research articles, incorporating rich media (video and podcasts), and so on. Most likely, at some point these innovations will become mainstream through increased introduction by traditional publishers, but then what? Stated another way, if we finally move away from the traditional PDF to something more dynamic that integrates data, rich media, and includes interactive access, what do I as a scientist want from publishers at that point?

To answer this question, let us start with where we are today. As authors, we put an enormous amount of effort into producing a publishable manuscript. At some point we pass it over to the publisher without a second thought. Subsequently, we will put a large amount of effort into a revision or rebuttal letter, but again, there is no thought on what will happen to our work after it has been accepted beyond the date it will be published and appear in PubMed. There is an enormous amount of trust in our publisher that our creations will be handled in the best possible way and, when published, that they will be disseminated to all who want to read our work. Open access introduced a hairline fracture in this trust with some scientists realizing that perhaps their work was not being as widely accessed as possible. Nevertheless, most scientists still do not think seriously about limited access and signing away the copyright. After all our efforts at producing a paper, very few of us have asked the question, is journal *x* presenting my work in a way that maximizes the understanding of what has been done, providing the means to ensure maximum reproducibility of what has been done, and maximizing the outreach of my work? I would suggest that now is the time not to just toss the paper over a high barrier to the journal and forget about it, but to break down the barrier and have a new form of interaction and dialog with a publisher who is prepared to embrace a changing publishing model and can answer the question in a satisfactory manner. In other words, we have an interaction with the publisher that does not begin when the scientific process ends, but begins at the beginning of the scientific process itself.

Perhaps you are beginning to see why I get so many blank stares when I raise this issue with scientists (producers and consumers) and publishers (service providers), but let me press on. Performing scientific research can be represented as a workflow. First, there is an idea that then is formulated as a hypothesis. An experiment is designed to test that hypothesis. The experiment produces data that are analyzed, generating results. Those results are discussed and conclusions drawn. Today, much of the product of that workflow is in digital form, and in the field of computational biology it may all be in digital form. Then comes the barrier that we climb over to publish. Everything we have done needs to be retrofitted to a medium that really does not represent our work in the best possible way. For example, the data from which the conclusions were drawn and the conclusions themselves may now be disjointed, perhaps presented in two separate public repositories (journal and database) with only a tenuous, if any, link between them. Much of the work may have to be omitted to meet restrictions imposed by page limits (or page charges) that do not really make sense in an electronic medium. Visualization of the data, which was so easily accomplished in the laboratory, is impossible in the final published article. *In summary, the final published work does not map well to the workflow of the scientific endeavor used to create it. In the digital era there is no excuse for not doing better*. The digital era transformed how science was disseminated and in so doing the word “paper” became synonymous with the term “PDF”—the same content just delivered differently. We are at a point where the word PDF will soon be replaced by something else; let's just call it an interactive PDF. What I am suggesting is that one day the interactive PDF will be replaced by the scientific workflow as the entity by which we get credit as scientists. The workflow will make science more reproducible and more open, and this is how I want the publisher of the future to handle my scientific output—*I want publishers to publish my workflows*. The notion of a workflow here is perhaps slightly different than that defined by many of this readership. It is less of a computational workflow, but part process and part container for content (or pointers to that content) that is significantly broader and more integrated than what is sent for publication today, namely, a manuscript and supplemental information in an essentially computationally unusable form.

There is synergy here with the idea of Open Notebook Science (http://en.wikipedia.org/wiki/Open_Notebook_Science), but there are also differences. Here, something is only open when the laboratory chooses to make it so, and so does not necessarily imply total openness, but I would guess that greater openness would result, if only by default. More importantly, open notebooks do not necessarily extend to publishing, but they could.

What are the incentives for moving in this new publishing direction? I would suggest that some incentive will come from scientists seeing this as an opportunity for their workflows to become more efficient and persistent, and, with publishing (aka recognition and availability) as the end product, will push to make this happen. Consider a few inefficiencies and persistence issues from my own current scientific workflow to make the point:

- The intellectual memory of my laboratory is in my e-mail folders, themselves not perfectly organized. This creates a hub-and-spoke environment where lab members and collaborators have to too often go through me to connect to each other.
- Much of our outreach is in the form of presentations made to each other and at national and international forums. We do not have a good central repository for this material; such a repository could enable us to have a better understanding of what other researchers are doing.
- While we endeavor to make all our software open source, there are always useful bits of code that languish and disappear when the author leaves the laboratory.
- Important data gets lost as students and postdoctoral fellows leave the laboratory.

I could go on embarrassing myself, but I think you get the picture. In a world of perfect workflows, I could immediately reconstruct the history of all work done in my laboratory from different viewpoints, for example by project, subproject, or scientist. At my fingertips I would have a preserved copy of all data, presentations, intellectual exchanges that have been undertaken, papers that have been read and studied, and so on.

So what does this have to do with publishers? I want the publisher of the future, or the publisher in collaboration with a third party, to be the guardian of these workflows in the same way that today I entrust them with the finished product of my research. The publisher becomes responsible for the whole kit and caboodle. Some would say that much of what is published today should not be, so why add more superfluous information to the record of science? The response is that one person's trash is another person's treasure. What is important is that the tools exist for a consumer to efficiently make their own judgment between the treasure and the trash. Those tools need to be able to navigate and summarize the workflows and in fact make associations that are just not possible today, but lead to new discoveries. There is a business model in what I propose since I think many of us would write into grants the cost of having a publisher, or third party in collaboration with the publisher, maintain our scholarly output in the way described. Presumably, funding agencies would fund such requests since it would make scientists more efficient and create a better scholarly record. Funders are already pushing in this direction of greater access to data and scientific papers, so this is an extension of that mandate. Those funds would be passed to the publisher of the future in the same way as open-access charges and page charges are today.

There are many issues with the concept of not publishing PDFs and publishing workflows instead. It is much harder to manage, for a start. The PDF is a single static interface that we all understand and can use. A workflow is more dynamic and can be viewed from a variety of perspectives in the same way a database or content management system presents multiple views of the content. This flexibility could be very powerful, but would represent a major change for most scientists. A change of work habit is only one major barrier to the workflow vision. There is something comforting about the simple organization of a paper and the relatively brief description of the work relative to what is proposed here. But really, is the work provided in our current scholarly discourse reproducible and can it be built upon? The manuscript also provides a creative medium through which authors can express themselves; there is a risk of losing this human element if too much structure is imposed. A counter view is that the workflow as content container could include audio and video discussions by the authors that would make the content potentially more accessible.

The scientific endeavor as a simple linear workflow is also clearly an oversimplification. The author needs to present components of the workflow that make sense and can be followed, rather than the endless iterations that happen in daily research, but that is not to say negative data and experiments should be excluded. Alternatively, the same experiments may result in more than one paper, and in this new paradigm parts of the workflow would be reused and hence not original. As long as this is declared, a complete workflow can still be judged for its originality. Expecting the publisher to manage the complete workflow may be too much. Perhaps the answer is to have a shared content model and subsequent easy navigation between publisher and institutional repositories where governance of the workflow is shared. Right now it seems the content of the respective repositories is either totally repetitive or not linked to publishers in any way. An alternative model might be to have a third party manage content as an intermediary between institution and publisher. Whereas a published paper is an end product, workflows (data, methods, etc.) are likely to continue to change, so versioning becomes important, but can be handled. Research funding agencies could, and should, promote these type of governance models and hence catalyze their adoption.

There are also the more recognizable issues, so let us consider a few of these:

- *Confidentiality*. The system that maintains the workflows can do this in the same way a journal management system handles the manuscript submission, peer review, and editorial process today. Specific individuals, groups, and the community at large can be provided appropriate levels of access to each element of the workflow at appropriate times. It is likely that much more of the scientific endeavor would be freely released to the community than happens today. Hopefully, this would accelerate scientific discovery worldwide. Proper attribution could be given by tagging components of the workflow so they can be attributed to their original source.
- *Peer Review*. Certainly this would be more demanding and tools would be needed to do a good review since it becomes more than just reading a paper, but exploring the workflow. There are instances already where publishers require the data so that the reviewers can truly evaluate the paper (e.g., some journals of the International Union of Crystallography); review of workflows takes this a step further.
- *New Infrastructure*. Publishers already provide Web-accessible servers for appropriate audiences to access manuscripts under review and final published papers. Commercial systems do exist for supporting workflows and managing projects today. These could be extended for the task of maintaining and publishing workflows, although few publishers would seem equipped to do this today.
- *Data Repositories versus Publishers*. Part of the workflow proposed, namely the data, may currently reside in public repositories with their own standards, reward system, politics, and so on; how can this be reconciled with the publishers presumed to take on this role? In the world of interoperability, cloud computing, and other buzz words, there is no reason why the workflow need reside all in one physical place; it just needs to appear that way to the user. Today publishers enable data repositories by insisting data associated with a publication are deposited therein. In the future, that contract would need to be expanded to provide more seamless interoperability that would seem to benefit everyone.
- *Community Acceptance*. At first glance, what is proposed for the publisher of tomorrow appears as a radical departure from what is done today; however, it can be done in stages. Consider much of what is published in this journal. It can be distilled to software (methods), data (supplementary material), and annotations (research articles). It is not a huge jump to imagine these integrated and accessible through an online interface. Other parts of the workflow could be integrated over time. Some publishers already provide repositories for other components of the workflow (e.g., Nature Precedings), but it is just not integrated with what is considered the final product today, namely the published PDF. A gradual change in a conservative marketplace would seem the most realistic. It also allows for gradual experimentation as to what the current research article interface can realistically morph into. There still needs to be a succinct summary of the workflow; will that be the research article or something else? There also needs to be an ongoing and accepted reward system by the community of scholars, otherwise it will not be adopted, even though sustainability alone is a compelling argument.
- *Journals and the Reward System*. The success of a scientist has traditionally been tied to the journals in which he or she publishes. In part, this arises because those who assess us do not intimately know our work and they use the quality of the journal as their guide. In some ways, this is very unscientific since reviewers are considering data from a whole journal, not the paper itself. Article-level metrics and the emerging interest in bibliometrics in an online world change this situation, raising issues associated with the journal concept itself. Publishing workflows versus publishing research articles is just another facet of this sea change that needs to be considered and value measured.

According to the *Fourth Paradigm*, computation will touch every aspect of the scientific endeavor. Organizations like Orwik (http://www.orwik.com/) and Mendeley (http://www.mendeley.com/) are already pushing in this direction, and the RSS system [5] focuses on reproducibility through workflows, all without the publishing focus. The complete result will be a digital workflow that begins with a documented idea and ends in a set of conclusions from a scientific experiment, all of which will be published by the publisher of the future and accepted as the norm in scholarly communication. Fact or fiction? Let us know what you think by using the comment feature associated with this article.
